# Intersecting Pathologies: A Case Report on the Co-presentation of Quasi-Moyamoya Disease and Polyneuropathy, Organomegaly, Endocrinopathy, Monoclonal Plasma Cell Disorder, and Skin Changes (POEMS) Syndrome

**DOI:** 10.7759/cureus.65934

**Published:** 2024-08-01

**Authors:** Jana N Satma, Iman Moradi, Lena Khachik, Gina Saad, Carlos Gonzalez

**Affiliations:** 1 Internal Medicine, St. George's University School of Medicine, St. George's, GRD; 2 Internal Medicine, Queens Hospital Center, Queens, USA; 3 College of Medicine, St. George's University School of Medicine, St. George's, GRD; 4 Internal Medicine, Icahn School of Medicine at Mount Sinai, New York, USA

**Keywords:** cerebral infarct, polyneuropathy, moyamoya disease, vasculopathy, vascular endothelial growth factor (vegf), quasi-moyamoya disease, poems syndrome

## Abstract

Moyamoya disease (MMD) is a rare chronic vasculopathy characterized by progressive stenosis of the internal carotid arteries and the formation of fragile collateral vessels in the brain. Polyneuropathy, organomegaly, endocrinopathy, monoclonal plasma cell disorder, and skin changes (POEMS) syndrome is a rare paraneoplastic syndrome with a complex presentation that includes polyneuropathy, organomegaly, endocrinopathy, M-protein, and skin changes. Here, we report a unique case of a 54-year-old male with MMD presenting with recurrent speech loss and mumbling, later diagnosed with POEMS syndrome. Initial imaging revealed Moyamoya vasculopathy, confirmed by computed tomographic angiography (CTA) and magnetic resonance imaging (MRI). Further examination revealed polyneuropathy, organomegaly, and elevated vascular endothelial growth factor (VEGF), meeting the diagnostic criteria for POEMS syndrome. The patient was treated with a cyclophosphamide-bortezomib-dexamethasone regimen, followed by the addition of daratumumab, resulting in clinical improvement. This case highlights the importance of thorough diagnostics and a multidisciplinary treatment approach for patients with complex comorbidities, emphasizing the need for early detection and targeted therapy in managing dual pathologies of MMD and POEMS syndrome.

## Introduction

Moyamoya disease (MMD) is a chronic vasculopathy characterized by progressive narrowing of the internal carotid arteries and their branches. It is associated with the formation of collateral vessels at the cerebrum that form in response to chronic brain ischemia. The term "Moyamoya" originates from Japanese, meaning "a puff of smoke," used to describe the appearance of the fragile network of newly formed vessels. Chronic brain ischemia is believed to induce an overexpression of proangiogenic factors, which promotes the formation of this vascular network. The exact cause of MMD remains unclear, although both genetic and acquired factors are thought to play a role. Mutations in the BRCC3/MTCP1 and GUCY1A3 genes have been reported in the literature, suggesting a genetic component to the disease [1]. Moyamoya syndrome (MMS) is characterized as Moyamoya disease occurring in conjunction with other conditions that include, but are not limited to, neurofibromatosis I, sickle cell disease, and Down syndrome. MMD commonly appears in two primary forms: ischemic strokes or hemorrhagic strokes, accompanied by potential symptoms such as seizures or headaches [2]. 

Polyneuropathy, organomegaly, endocrinopathy, monoclonal plasma cell disorder, and skin changes (POEMS) syndrome is a rare paraneoplastic syndrome caused by plasma cell proliferation and characterized by the presence of polyneuropathy, organomegaly, endocrinopathy, M-protein, and skin changes. Its diagnosis is based on the current Dispenzieri diagnostic criteria, which requires mandatory criteria, including a polyneuropathy and monoclonal plasma cell-proliferative disorder and at least one major and one minor criterion [3]. Major criteria include sclerotic bone lesions, elevated vascular endothelial growth factor (VEGF), and Castleman disease. Minor features include organomegaly, endocrinopathy, skin changes, papilledema, extravascular volume overload, and thrombocytosis [4].

Cases linking MMD with POEMS syndrome are exceptionally rare, with existing data documented only in the form of a few case reports. Herein, we report a unique case of a patient with MMD who presented with an initial manifestation of speech loss and mumbling and later presented with features of POEMS syndrome. This case emphasizes the importance of thorough diagnostics to ensure correct treatment approaches in patients with complex comorbidities.

## Case presentation

The patient is a 54-year-old male with a past medical history of peripheral neuropathy, polysubstance use disorder (marijuana and cocaine), protein S deficiency, prior splenic infarct, and a prior deep venous thrombosis. The patient presented to the primary care physician with complaints of recurrent episodes of speech loss and mumbling for the past two weeks. The episodes usually last for 15-20 minutes before they fully resolve, leaving no residual symptoms. His last episode resulted in a fall and shaking of the upper extremities with no loss of consciousness or incontinence, which led him to visit the emergency department (ED). In the ED, a stroke was suspected, and an appropriate workup was performed. Initial physical examination was positive for sensory changes, speech changes, and focal weakness.

**Figure 1:**
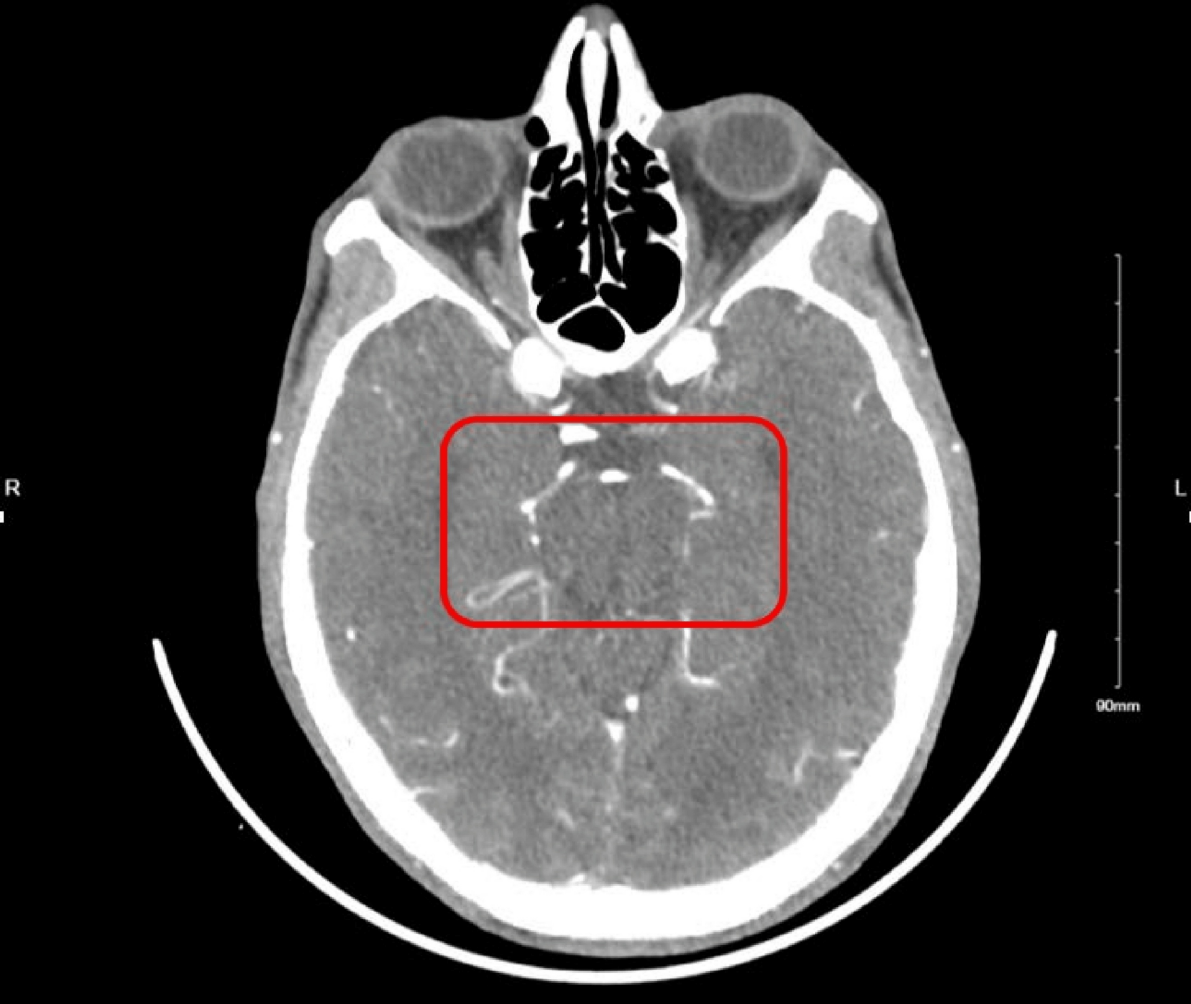
Bilateral attenuated cavernous internal carotid arteries with diminutive M1 segments. Small vessel caliber and decreased enhancement extends into the right M2 segments.

**Figure 2:**
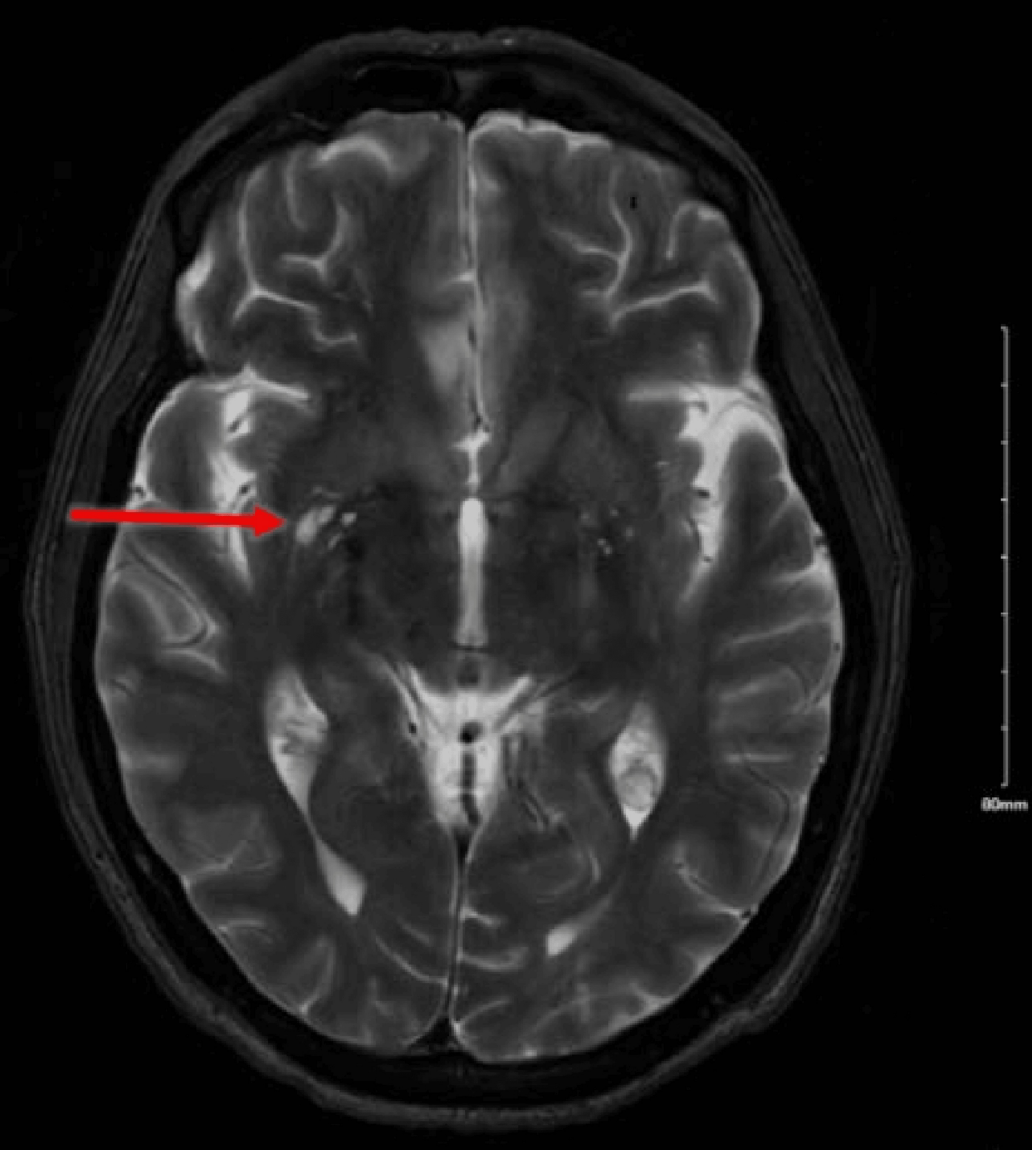
Worsening bilateral internal border zone infarcts.

Computed tomographic angiography (CTA) of the head demonstrated bilateral attenuated cavernous internal carotid arteries with diminutive M1 segments. Small vessel caliber and decreased enhancement extends into the right M2 segments (Figure 1). Magnetic resonance imaging (MRI) of the brain showed acute infarcts in the left posterior border zone territory and scant punctate infarcts in the right hemisphere (Figure 2). Fluid-attenuated inversion recovery and MRI demonstrated hyperintensity of vessels on the cortical surface and slow flow in the bilateral distal middle cerebral artery (MCA) territory. Angio-cerebral perfusion with Omnipaque suggested a 101-mL volume of hypoperfusion in the frontal lobes, without a core infarction being identified. This distribution largely agreed with diffusion-weighted imaging MRI. These findings were consistent with Moyamoya vasculopathy. He was then discharged, his medication was refilled, and he was scheduled to follow up with a neurology outpatient.

**Figure 3:**
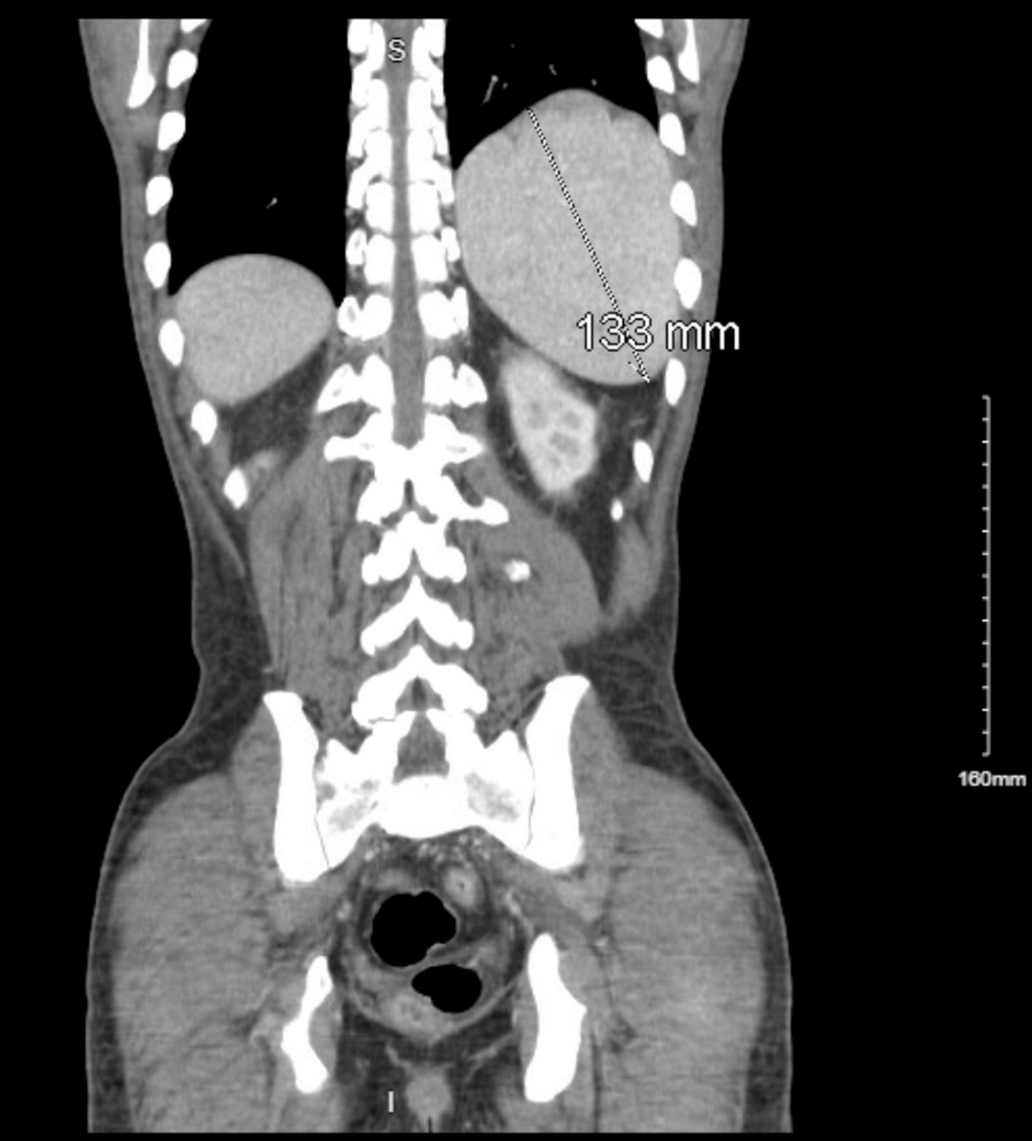
Splenomegaly with mildly enlarged retroperitoneal and pelvic lymph nodes.

**Figure 4:**
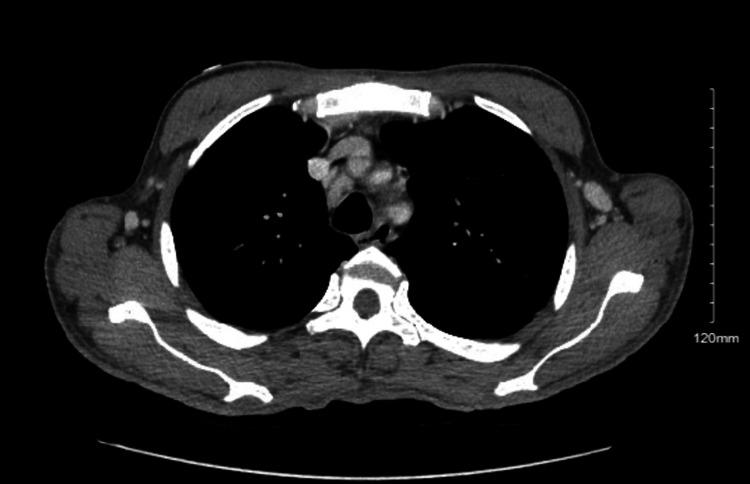
Multifocal thoracic lymphadenopathy, including axillary, supraclavicular, subpectoral, mediastinal, and hilar lymphadenopathy. Hepatosplenomegaly is also present.

The patient lost follow-up for three months, after which he presented with transient expressive aphasia. He appeared cachectic and disheveled, with a 15 lb weight loss over the past three months. Physical examination was additionally significant for testicular atrophy and generalized lymphadenopathy. Chest and abdominal computed tomography (CT) scans showed lymphadenopathy as well as splenomegaly (Figures 3, 4).

Further investigation revealed that the patient now has POEMS syndrome, meeting several diagnostic criteria: the presence of a monoclonal gammopathy characterized by two IgA lambda bands, peripheral neuropathy evidenced by an electromyogram showing diffuse moderate sensorimotor axonal and demyelinating peripheral polyneuropathy affecting both lower extremities, and Castleman's disease confirmed by an inguinal lymph node biopsy. Additional diagnostic indicators include elevated vascular endothelial growth factors (VEGFs), organomegaly (specifically splenomegaly), endocrinopathy involving thyroid and gonadal dysfunction, elevated protein levels in the cerebrospinal fluid without significant cellularity, thrombocytosis, and thrombotic diathesis marked by a history of splenic infarcts, cerebral infarcts, and lower extremity deep vein thrombosis. The patient also exhibited other symptoms and signs, such as skin changes, weight loss, and fatigue.

Following the confirmation of the diagnosis, treatment was initiated with cyclophosphamide-bortezomib-dexamethasone (CyBorD), accompanied by regular hematologic monitoring. The patient was placed on a weekly regimen consisting of 1.3 mg/m² of bortezomib, 300 mg/m² of cyclophosphamide, and 40 mg of dexamethasone. This regimen was administered for four weeks, resulting in an overall improvement in the patient's clinical presentation with only mild side effects, including vitamin B6 deficiency, which was managed with a daily dose of 100 mg of vitamin B6. In the fifth week of treatment, weekly infusions of daratumumab were added to the regimen. Neurology recommended the reinitiation of 81 mg of aspirin daily and 40 mg of atorvastatin daily.

## Discussion

The patient in question presented with typical features of Moyamoya disease; however, further workup led to an additional diagnosis of POEMS syndrome. This co-presentation is particularly noteworthy given that both disorders predispose to vascular abnormalities: vascular narrowing and occlusion in Moyamoya disease and a prothrombotic state in POEMS syndrome.

Quasi-Moyamoya disease and Moyamoya disease share similarities in their presentation but are distinguished by their underlying etiologies. Moyamoya disease is characterized by chronic progressive stenosis of the terminal portion of the internal carotid arteries, leading to the development of an abnormal vascular network at the base of the brain. This idiopathic condition often results in ischemic strokes or hemorrhages due to the compromised blood flow and formation of collateral vessels known as Moyamoya vessels [5]. In contrast, quasi-Moyamoya disease presents with similar vascular abnormalities but is secondary to other underlying conditions such as atherosclerosis, autoimmune diseases, or inflammation. Unlike Moyamoya disease, quasi-Moyamoya disease’s stenotic changes often have an identifiable cause, distinguishing it from the idiopathic nature of Moyamoya disease [5,6]. While both conditions involve progressive vascular occlusion and the development of collateral networks, quasi-Moyamoya is frequently linked to systemic diseases or prior radiation therapy, making it a heterogeneous group of conditions rather than a single distinct disease [5,7].

Cases have been documented where patients with POEMS syndrome develop quasi-Moyamoya disease, which involves stenosis or occlusion of major cerebral arteries accompanied by an abnormal vascular network [6,7]. Elevated VEGF levels in POEMS syndrome induce angiogenesis and increased vascular permeability, which can lead to the development of Moyamoya vessels, potentially resulting in ischemic strokes or intracranial hemorrhages. Surgical interventions, such as cerebral revascularization, have shown promise in managing ischemic strokes associated with this dual pathology, highlighting the need for early and aggressive treatment strategies to mitigate poor prognostic outcomes [7].

Unexpectedly, the patient's condition progressed in unexpected ways, revealing new complexities. For instance, despite the recognized risk of hemorrhagic stroke in Moyamoya disease due to the fragility of collateral vessels, our patient primarily exhibited ischemic strokes. This could be suggestive of an atypical manifestation or an interaction between the proangiogenic milieu driven by high VEGF levels in POEMS syndrome and the intrinsic vascular pathology of Moyamoya disease. Elevated VEGF is a hallmark of POEMS and is known to contribute to endothelial proliferation and vessel permeability, potentially exacerbating the angiogenic drive in quasi-Moyamoya disease [6,8].

The management strategy adopted for our patient, involving immunomodulatory therapy alongside standard vascular protective measures such as antiplatelet therapy, underscores the importance of targeting both the hematologic abnormalities intrinsic to POEMS syndrome and the vascular instability associated with Moyamoya disease.

## Conclusions

This case report presents a rare co-occurrence of Moyamoya disease and POEMS syndrome in a 54-year-old male, underscoring the complex interplay between these two conditions. The patient's initial presentation with speech loss and subsequent discovery of polyneuropathy, organomegaly, and elevated VEGF levels led to the dual diagnosis. The management strategy involving immunomodulatory therapy and vascular protective measures demonstrated clinical improvement, highlighting the importance of a comprehensive diagnostic and therapeutic approach. Early detection and targeted treatment are crucial in managing the overlapping vascular and hematologic abnormalities in patients with such complex comorbidities. This case underscores the need for awareness and further research into the interplay between MMD and POEMS syndrome to optimize patient outcomes. 
